# A Cloned Recombinant Vesicular Stomatitis Virus-Vectored Marburg Vaccine, PHV01, Protects Guinea Pigs from Lethal Marburg Virus Disease

**DOI:** 10.3390/vaccines10071004

**Published:** 2022-06-23

**Authors:** Wenjun Zhu, Guodong Liu, Wenguang Cao, Shihua He, Anders Leung, Ute Ströher, Michael J. Fairchild, Rick Nichols, Joseph Crowell, Joan Fusco, Logan Banadyga

**Affiliations:** 1 Special Pathogens Program, National Microbiology Laboratory, Public Health Agency of Canada, Winnipeg, MB R3E 3R2, Canada; wenjun.zhu@inspection.gc.ca (W.Z.); guodong.liu@phac-aspc.gc.ca (G.L.); wenguang.cao@phac-aspc.gc.ca (W.C.); shihua.he@phac-aspc.gc.ca (S.H.); anders.leung@phac-aspc.gc.ca (A.L.); stroheru@who.int (U.S.); michael.fairchild@childrens.harvard.edu (M.J.F.); 2 Public Health Vaccines, Cambridge, MA 02142, USA; rnichols@phvaccines.com (R.N.); jcrowell@phvaccines.com (J.C.); jfusco@phvaccines.com (J.F.)

**Keywords:** Marburg virus, VSV-MARV, vesicular stomatitis virus, vaccine, filovirus

## Abstract

Marburg virus (MARV) is a negative-sense, single-stranded RNA virus that belongs to the *Filoviridae* family. Despite having caused numerous outbreaks of severe hemorrhagic fever with high case fatality rates, there are still no clinically approved therapeutics or vaccines to treat or prevent MARV disease. Recombinant vesicular stomatitis viruses (rVSVs) expressing heterologous viral glycoproteins have shown remarkable promise as live-attenuated vaccine vectors, with an rVSV-based Ebola virus vaccine having received regulatory approval in the United States and numerous other countries. Analogous rVSV vaccine vectors have also been developed for MARV and have shown efficacy in several preclinical studies conducted in nonhuman primates. Here, we used a guinea pig model to confirm the protective efficacy of a cloned, rVSV-based candidate vaccine, termed PHV01, expressing the MARV variant Angola glycoprotein. Our results demonstrated that a single dose (2 × 10^6^ PFU) of vaccine administered 28 days prior to challenge with a uniformly lethal dose of guinea-pig-adapted MARV variant Angola provided complete protection from death and disease. Moreover, protection was robust, with as little as 200 PFU of vaccine conferring significant protection. Not only does this study highlight the potential predictive value of the guinea pig model in the evaluation of MARV countermeasures, but it also demonstrates consistent and reproducible protection afforded by a clonal vaccine candidate. Indeed, this study identifies PHV01 as a suitable vaccine candidate for advanced development.

## 1. Introduction

Marburg virus (MARV)—like the related Ebola virus (EBOV)—is a negative-sense RNA virus that belongs to the family *Filoviridae* [1]. Like EBOV, MARV causes severe viral hemorrhagic fever in humans, with high case fatality rates. Clinically, Marburg virus disease (MVD) is characterized by influenza-like symptoms and a high fever that progresses rapidly towards gastrointestinal and neurological dysfunction, as well as blood coagulation abnormalities and hemorrhagic manifestations [2,3]. This pathology seems to be driven by the damage caused by rampant and systemic virus replication in combination with an excessive inflammatory response. In severe cases, death results from multiple organ dysfunction and shock.

Since the discovery of MARV over 50 years ago, there have been 14 recorded outbreaks of MVD, with an overall case fatality rate of ~81% [1]. The largest and most severe outbreak occurred in Angola between 2004 and 2005, resulting in 252 cases and 227 deaths [4]. The Angola variant of MARV (MARV/Ang) is derived from this outbreak and thought to be among the most virulent of all MARV variants [5]. Notably, a single fatal case of MVD was recently identified in Guinea [6], making this the first report of MARV in a human in West Africa and underscoring the threat that this virus poses to public health.

The World Health Organization lists MVD as one of a few priority diseases requiring urgent research and development attention [7]. Yet, despite the continuous threat of MARV outbreaks, there are still no licensed vaccines or approved countermeasures for MVD. Nevertheless, several vaccine candidates have been developed and many have shown promising efficacy in various animal models [8]. Among the most promising vaccine candidates are those based on the recombinant vesicular stomatitis virus (rVSV) vector platform. In general, two approaches have been taken to generate replicating rVSV-based MARV vaccines that elicit a potent and protective immune response directed against the MARV glycoprotein (GP), the viral protein responsible for virion entry and fusion [3]. In the first approach, the native VSV glycoprotein (G) gene is replaced with the MARV GP gene to generate live-attenuated vaccines broadly referred to as rVSVΔG-MARV [9]. An analogous rVSV-based vaccine against EBOV has proven highly efficacious and was recently approved for clinical use by the US FDA [10]. In the second approach, the MARV GP gene is inserted into a VSV vector with a rearranged gene order that retains expression of a truncated VSV G; this live-attenuated vaccine is referred to as rVSVN4CT1-MARV [11].

Preclinical work in nonhuman primates (NHPs) has demonstrated that a single dose of rVSVΔG-MARV expressing GP from the MARV variant Musoke (MARV/Mus) confers 100% protection against challenge with the homologous virus [12], offering durable immunity lasting at least 14 months [13] and an exceptional safety profile [14]. Post-exposure efficacy has also been demonstrated using this vaccine, with 100% survival in animals vaccinated 30 min after infection with MARV/Mus [15], 83% survival in animals vaccinated after 24 h [16], and 33% survival in animals vaccinated after 48 h [16]. Although the MARV/Mus-based vaccine is effective in NHPs challenged with MARV/Ang or the related Ravn virus [17], the efficacy of a MARV/Ang-based vaccine and MARV/Ang challenge remains relatively understudied. To date, one study has demonstrated complete protection in NHPs immunized with an rVSVΔG-MARV vaccine expressing MARV/Ang GP and challenged with MARV/Ang [18]. A follow-up study in the same NHP model demonstrated the ability of this vaccine to confer rapid protection, with 100% survival following challenge (1000 PFU, IM) as early as 7 days postvaccination and 75% survival at 3 days postvaccination [19]. These results highlight the potential of this vaccine for rapid intervention in an outbreak setting. Interestingly, a separate study showed limited postexposure efficacy in NHPs, with only 25% of animals surviving when vaccinated 20–30 min after a high-dose challenge of MARV/Ang [20] in contrast to the results observed for MARV/Mus challenge [12]. Postexposure protection increased to 75% when a lower dose of MARV/Ang was used for challenge [20]. Notably, the rVSVN4CT1-MARV vaccine expressing MARV/Ang GP also showed effective postexposure protection against a low dose (50 PFU, IM) of MARV/Ang, with 60% of animals surviving challenge [20]. The same rVSVN4CT1-MARV vaccine has also been shown to protect NHPs against MARV/Ang (1000 PFU, IM) when administered as part of a trivalent (EBOV, MARV, and Sudan virus) filovirus vaccine [11]. Advanced development of these MARV vaccine candidates is now progressing, as preparedness to counteract the threat of emerging diseases of epidemic or pandemic potential has become an urgent priority [21].

In this study, we sought to advance the preclinical development of an rVSVΔG-MARV/Ang vaccine termed PHV01. In particular, we evaluated three vaccine virus materials: (1) an uncloned research virus stock at passage level 3 (rVSVΔG-MARV P3); (2) a cloned (i.e., plaque-purified) GMP-produced Master Viral Seed (PHV01 MVS) at passage level 9; and (3) a GMP-produced cloned Formulated Drug Substance (PHV01 FDS) at passage level 11 (Figure S1). The vaccine materials were tested in the well-defined guinea pig model, which uses guinea-pig-adapted MARV/Ang (GPA-MARV/Ang) as the challenge virus to produce a uniformly lethal infection that recapitulates several hallmarks of MVD, including lymphocytopenia, thrombocytopenia, high viremia, and systemic spread of virus [22]. Following immunization with a single 2 × 10^6^ PFU dose, all three vaccines completely protected guinea pigs from lethal MARV challenge. A dose level of 2 × 10^4^ PFU conferred complete or partial protection, depending on the vaccine material used, and a dose level of 2 × 10^2^ PFU conferred significant but partial protection. Not only does this study further demonstrate the protective efficacy of the rVSV-based MARV vaccine, but it also specifically demonstrates the efficacy of the PHV01 FDS vaccine clone, which was produced via a representative clinical GMP manufacturing process. The results presented here are important to the ongoing development of this promising vaccine candidate and a step towards achieving a safe and effective single-dose vaccine capable of preventing MVD.

## 2. Materials and Methods

### 2.1. Animal Ethics and Biosafety Statement

Animal experiments were reviewed and approved by the Animal Care Committee of the Canadian Science Centre for Human and Animal Health (CSCHAH), Winnipeg, Manitoba, in accordance with guidelines from the Canadian Council on Animal Care. All staff working on animal experiments completed education and training programs according to the standard protocols appropriate for this level of biosafety. All work with infectious MARV was performed in the containment level (CL)-4 laboratories at the CSCHAH in accordance with standard operating protocols.

### 2.2. Viruses and Cells

Vero E6 cells were obtained from the American Type Culture Collection and maintained at 37 °C and 5% CO2 in Dulbecco’s modified Eagle medium (DMEM; HyClone) supplemented with 5% heat-inactivated fetal bovine serum (HI-FBS; HyClone), 2 mM L-glutamine (HyClone), 50 U/mL penicillin, and 50 ug/mL streptomycin (HyClone). Guinea-pig-adapted Marburg virus variant Angola (GPA-MARV/Ang; Marburg virus/NML/C.porcellus-lab/AGO/2005/Ang-GA-P2; GenBank accession no. MF939097) was generated as previously described [22].

### 2.3. VSV-Based Vaccines

This study evaluated the protective efficacy of a recombinant vesicular stomatitis virus (rVSVΔG)-based, live attenuated vaccine against MARV. This vaccine, referred to as PHV01, expresses the MARV variant Angola glycoprotein (GP) in place of the VSV glycoprotein (G). The virus was generated via reverse genetics rescue essentially as described previously for a similar construct expressing the MARV variant Musoke GP [12]. A passage level 2 (P2) stock of virus was derived from the rescued virus (considered P0) via amplification on Vero E6 cells at the Public Health Agency of Canada. The P2 stock virus was subsequently amplified and plaque purified in qualified CGMP Vero cells in proprietary serum-free tissue culture media to generate premaster viral seeds at P8. A single P8 clone (Clone 5) was chosen based on plaque morphology, growth kinetics and productivity in Vero cells, and MARV GP transgene sequence fidelity. This clone was amplified to P9 under CGMP conditions to generate the PHV01 MVS. PHV01 MVS release criteria included the well-defined requirements for GMP viral seed manufacturing for purity (including adventitious agents), potency/strength, identity, and sterility. PHV01 MVS was further amplified to P10 under CGMP conditions to generate the PHV01 Working Viral Seed (WVS). The PHV01 Formulated Drug Substance (FDS) at P11 was produced from the PHV01 WVS in a disposable bioreactor using the same CGMP Vero cells and proprietary media described above. PHV01 FDS was harvested from cell culture medium containing virus, clarified, and further purified by nuclease digestion, depth filtration, and tangential flow ultrafiltration/diafiltration in a recombinant human albumin and Tris buffer formulation. Separately, an rVSVΔG-MARV P3 virus was generated from the initial P2 stock virus for research purposes, and PHV01 MVS was passaged three additional times (PHV01 MVS+3) to P12 (Figure S1).

Next-generation sequencing was used to confirm the sequence identities of the following viruses: the uncloned P2 stock virus, the premaster viral seeds (P8), PHV01 MVS (P9), PHV01 WVS (P10), and PHV01 MVS+3 (P12). In particular, PHV01 MVS and PHV01 WVS demonstrated 100% and 99.99% identity to the full-length genome plasmid used to rescue the virus, and no high-frequency mutations were identified in the MARV GP coding sequence. These data also highlight the exceptional stability of the rVSV genome over multiple passages (Figure S1).

The following vaccines were evaluated in this study: rVSVΔG-MARV P3, PHV01 MVS (P9), and PHV01 FDS (P11).

### 2.4. Guinea Pig Study Design

This study was composed of two experiments, termed Experiment #1 and Experiment #2. Both were designed to test the ability of PHV01 to protect female Hartley guinea pigs (Charles River Laboratories) from morbidity and mortality resulting from inoculation with a lethal dose of GPA-MARV/Ang. In Experiment #1, rVSVΔG-MARV research virus P3 and PHV01 MVS were evaluated at three different dose levels: 2 x 10^6^ plaque-forming units (PFU; high), 2 × 10^4^ PFU (medium), and 2 × 10^2^ PFU (low). Groups of 6 guinea pigs were immunized with one of the two vaccine preparations at one of the three specified doses (prepared with sterile, nontoxic, nonpyrogenic 0.9% saline as diluent), while a control group of 6 animals received an equivalent volume of 0.9% saline. In Experiment #2, the PHV01 FDS was evaluated at two different doses, high and medium (as described above), and PHV01 MVS was evaluated again at the high dose for comparative purposes. Groups of 6 guinea pigs were immunized with one of the two vaccine preparations at the specified doses (as described above), and a control group of 6 animals receiving 0.9% saline was also included. All vaccines were administered intramuscularly (IM) in a total volume of 300 µL, with 150 µL delivered to each of the rear quadriceps muscles. Twenty-eight (28) days postvaccination (DPV), animals were inoculated with 1000 times the median lethal dose (LD_50_) of GPA-MARV/Ang via intraperitoneal (IP) injection. Animals were monitored for disease and survival up to 29 days postinfection (DPI), equivalent to 57 DPV in Experiment #1 and up to 28 DPI (56 DPV) in Experiment #2. EDTA blood and plasma (Experiment #1) or serum (Experiment #2) samples were obtained at 0 DPV (prior to vaccination), 2 DPV, 27 DPV, 5 DPI (33 DPV), and 29 or 28 DPI (57 or 56 DPV).

### 2.5. VSV and MARV RNA Quantification

rVSVΔG-MARV (P3, PHV01 MVS, and PHV01 FDS) and MARV RNA were extracted from EDTA blood samples using the QIAamp viral RNA minikit (Qiagen) according to the manufacturer’s instructions (Qiagen). Viral RNA levels were determined by reverse transcription quantitative PCR (RT-qPCR) using the LightCycler 480 thermal cycler (Roche) and the LightCycler 480 RNA Master Hydrolysis Probes kit (Roche), along with the following primers and probe specific for MARV GP: forward primer, 5′-GTRTGCTCCGGRACYCTCCA-3′; reverse primer, 5′-YTGCCCRCTCAGTGTRAATC-3′; probe, 5′-6-FAM-RAARACAGA/ZEN/AGAYGTYCATCTGATGG-IABkFQ-3′. Cycling conditions were as follows: 63 °C for 3 min and 95 °C for 30 s, followed by 45 cycles of 95 °C for 15 s and 60 °C for 30 s. RT-qPCR cycle threshold (Ct) values were converted to genome equivalents per milliliter (GEQ/mL) using a standard curve generated from a plasmid containing the MARV genome.

### 2.6. IgG ELISAs

MARV GP-specific IgG was quantified in Lithium–heparin plasma (Experiment #1) or serum (Experiment #2) samples by indirect ELISA. Half-area high-binding 96-well assay plates (Corning) were coated using recombinant MARV GP protein with the transmembrane domain deleted (IBT, Cat. No. 0506-015) prepared in pH 9.5 carbonate–bicarbonate buffer (30 ng/well) at 4 °C overnight. After removing the coating solution, plates were incubated with 5% skim milk (BD Biosciences) prepared in PBS for 1 h at 37 °C. Serial dilutions of plasma or serum samples prepared in 2% skim milk in PBS were then applied to the plates at 4 °C overnight. Following washes with 0.1% Tween-20 in PBS, plates were incubated for 1 h at 37 °C with an HRP-conjugated goat anti-guinea pig IgG (H+L) secondary antibody (KPL, Cat. No. 14-17-06) diluted to 1:1000 in 2% skim milk in PBS. After an additional set of washes with 0.1% Tween-20 in PBS, the plates were incubated in TMB solution (Life Technologies) for ~30 min in darkness before optical density (OD) signals were measured at 650 nm using a Synergy HTX plate reader (Biotek). Endpoint dilution titers were calculated by determining the highest dilution that gave an average OD 650 reading greater than or equal to the cut-off OD value. The cut-off value was set per plate and calculated as the mean OD value for all 0 DPV samples (at dilutions of 1:200 or 1:400, depending on the assay) plus three times the standard deviation of that mean. In instances where the endpoint titer was determined to lie below the lower limit of detection or above the upper limit of detection, arbitrary values of 100 and 583,200 were assigned, respectively. Data are depicted as the Log_10_ of the reciprocal endpoint dilution.

### 2.7. Plaque Reduction Neutralization Test (PRNT)

Lithium–heparin plasma samples from Experiment #1 or serum samples from Experiment #2 were obtained at 27 DPV, heat-inactivated (60 min at 56 °C), spun down at 15,000× *g* for 5 min, and subjected to a plaque reduction neutralization test (PRNT) using PHV01 FDS, which had undergone a single freeze/thaw cycle. Plasma or serum samples were serially diluted twofold (starting at 1:40) in DMEM. PHV01 FDS virus was then diluted in DMEM to 667 PFU/mL, and 200 µL was added to an equivalent volume of diluted plasma or serum, for a total volume of 400 µL containing 133 PFU virus and final plasma/serum concentrations of 1:80, 1:160, 1:320, 1:640, 1:1280, and 1:2560. The virus and plasma/serum mixture was incubated for 1 h at 37 °C, after which 150 µL (containing 50 PFU virus) was added to each well of a 12-well plate containing confluent monolayers of Vero E6 cells. Each dilution was tested in duplicate, and DMEM-only was used as a negative control. At 3 DPI, cells were fixed in 10% formalin containing crystal violet (0.5% *w*/*v*). Plaques were counted manually and the percent inhibition of infection was calculated by dividing the number of plaques in each test sample by the number of plaques in the negative control. The highest dilution that resulted in ≥50% inhibition of infection (PRNT_50_) was determined. In instances where the PRNT_50_ titer was determined to lie below the lower limit of detection (i.e., 80) or above the upper limit of detection (i.e., 2560), arbitrary values of 40 and 5120 were assigned, respectively. Data are depicted as the Log10 of the PRNT_50_ titer.

### 2.8. Statistical Analyses

All graphs were generated using GraphPad Prism version 9; all statistical tests were performed using the same software. Statistical comparisons of the Kaplan–Meier survival curves were performed using the Log-Rank test with the Bonferroni correction for multiple comparisons. The ordinary one-way ANOVA test and Tukey’s multiple comparison test were used to compare group means or geometric means against all other group means within Experiment #1, Experiment #2, or the pooled data. P-values less than or equal to 0.05 were marked with one asterisk (*), less than or equal to 0.01 were marked with two asterisks (**), less than or equal to 0.001 were marked with three asterisks (***), and less than or equal to 0.0001 were marked with four asterisks (****). Nonsignificant statistical comparisons are not labelled.

## 3. Results

### 3.1. Vaccination with PHV01 Protects Guinea Pigs from Lethal GPA-MARV/Ang Infection

**Figure 1:**
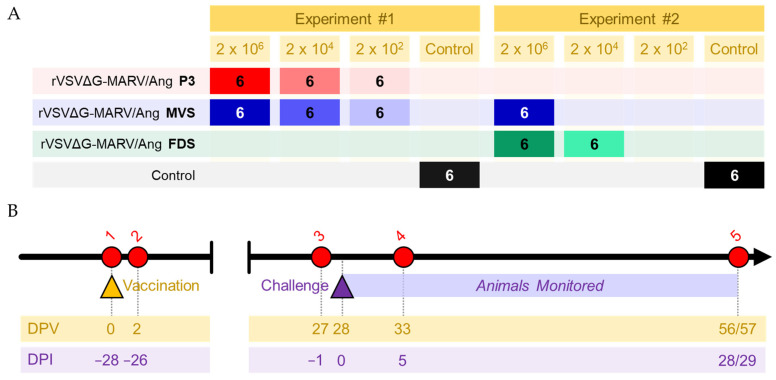
Study outline. (**A**) Groups of six guinea pigs were vaccinated with rVSVΔG-MARV P3, PHV01 MVS, or PHV01 FDS at one of three doses, 2 × 10^6^ PFU (high), 2 × 10^4^ PFU (medium), or 2 × 10^2^ PFU (low). Control animals were injected with an equal volume of 0.9% saline. The study was conducted in two independent experiments, with rVSVΔG-MARV P3 and PHV01 MVS evaluated in Experiment #1 and PHV01 MVS and PHV01 FDS evaluated in Experiment #2. (**B**) Twenty-eight days later, all animals were challenged with 1000 LD_50_ guinea-pig-adapted MARV variant Angola and monitored for signs of disease. Blood samples were obtained from each animal at the following time points: (1) prior to vaccination at 0 days postvaccination (DPV) or −28 days post-infection (DPI); (2) 2 DPV or −26 DPI; (3) 27 DPV or −1 DPI; (4) 33 DPV or 5 DPI; (5) 56/57 DPV or 28/29 DPI.

To assess the protective efficacy of rVSVΔG-MARV P3, PHV01 MVS, and PHV01 FDS, groups of six guinea pigs were immunized with varying dose levels of vaccine (high, 2 × 10^6^ PFU; medium, 2 × 10^4^ PFU; or low, 2 × 10^2^ PFU) and challenged 28 days later with a 1000 LD_50_ dose of GPA-MARV/Ang (Figure 1). rVSVΔG-MARV P3 and PHV01 MVS were evaluated in one experiment, while PHV01 FDS was evaluated in a second experiment (along with a repeat of the PHV01 MVS high dose level for comparison). None of the vaccinated animals exhibited any signs of morbidity following immunization, corroborating the high degree of safety associated with the VSV vaccine vector [23].

**Figure 2:**
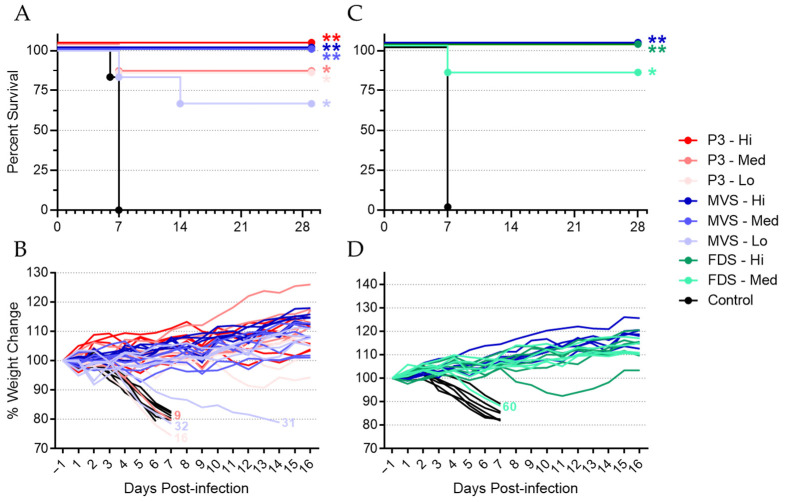
PHV01 prevents death and disease following MARV challenge. In Experiment #1 (**A**,**B**), guinea pigs (n = 6) were vaccinated with rVSVΔG-MARV P3 and PHV01 MVS at one of three different dose levels (high, medium, or low). In Experiment #2 (**C**,**D**), guinea pigs (n = 6) were vaccinated with PHV01 FDS at one of two different dose levels (high or medium) or with PHV01 MVS at the high dose level. Control animals (n = 6) in both experiments received saline. Twenty-eight days after vaccination, animals were challenged with a lethal dose of guinea-pig-adapted MARV and monitored for survival (**A**,**C**) and weight change (**B**,**D**). Survival curves for all vaccinated groups were significantly different from the control groups (Log-Rank with Bonferroni correction; *, *p* ≤ 0.05; **, *p* ≤ 0.01). Vaccinated animals that did not survive infection are indicated on the weight loss curves by their respective animal numbers: 9, rVSVΔG-MARV P3 medium dose; 16, P3 low dose; 31 and 32, PHV01 MVS low dose; 60, PHV01 FDS medium dose.

In general, vaccination with rVSVΔG-MARV P3 and PHV01 prevented severe disease and death due to MARV infection in nearly all animals (Figure 2). Animals vaccinated with the high dose of rVSVΔG-MARV P3, PHV01 MVS, or PHV01 FDS showed 100% protection from MARV, as did animals vaccinated with the medium dose of PHV01 MVS (Figure 2A,C and Table S1). The medium and low dose of rVSV-MARV P3, as well as the medium dose of PHV01 FDS, resulted in 83% survival, with one of six guinea pigs in each group (animals 9, 16, and 60) succumbing to MARV infection. The low dose of PHV01 MVS resulted in 67% survival, with two of six guinea pigs (animals 31 and 32) succumbing to infection (Figure 2A,C and Table S1). Notably, animal 9, in the rVSVΔG-MARV P3 medium-dose group, did not receive the full dose of vaccine, since a small volume of the dose was expelled from the quadriceps muscle after administration. We suspect that this technical error accounts for the fact that this animal did not survive GPA-MARV/Ang infection. All unvaccinated control animals succumbed to MARV infection by 7 DPI, and statistical analyses revealed that the survival curves for all vaccinated animals were significantly different from the survival curves for the control animals (Figure 2A,C).

As expected, all vaccinated, surviving animals gained weight following GPA-MARV/Ang infection (Figure 2B,D), and the vast majority exhibited no clinical signs of disease. Indeed, only 3 of the 32 vaccinated, surviving animals showed any clinical signs, which were all extremely mild (e.g., minor weight loss, ruffling of fur, and/or moderately reduced activity) and resolved completely within a few days and before 14 DPI. In contrast, almost all control animals, as well as the vaccinated, non-surviving animals, showed rapid weight loss (Figure 2B,D) and severe signs of disease (e.g., weight loss greater than 20%, very ruffled fur, hunched posture, and/or significantly reduced activity). Interestingly, animal 31, in the PHV01 MVS low-dose group, experienced a slowly progressive disease that culminated in euthanasia at 14 DPI (due to significant weight loss), much later than all other non-surviving animals (Figure 2A).

In total, of the 54 animals that were vaccinated with any vaccine, 49 survived GPA-MARV/Ang challenge, giving an overall vaccine efficacy of ~91% (Table S1). To specifically assess the level of protection conferred by the clonal PHV01, we pooled the data from the PHV01 MVS and FDS medium- and high-dose groups (Table S2). All animals (n = 18) that received the high dose of these vaccines survived, while only 1 of 12 animals that received the medium dose succumbed. Of the 30 animals vaccinated with either the high or medium dose of PHV01 MVS or FDS, 29 survived, giving an overall PHV01 vaccine efficacy of 97%.

### 3.2. Vaccinated Animals Exhibited Transient Vaccinemia

**Figure 3:**
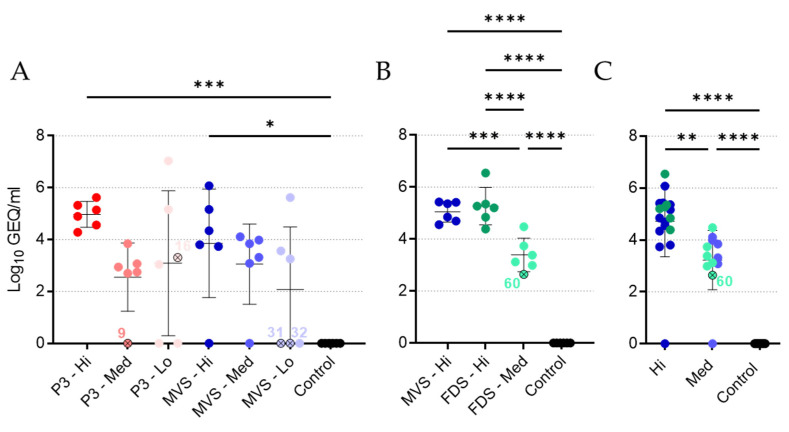
Vaccinemia following vaccination. Blood samples obtained from all guinea pigs at 2 days postvaccination (DPV) in Experiment #1 (**A**) or Experiment #2 (**B**) were assessed for levels of virus RNA via RT-qPCR. Data are presented as Log_10_ genome equivalents (GEQ) per milliliter, with means and standard deviations for each group depicted behind individual values for each animal. Data from the high and medium dose levels for the PHV01 MVS and PHV01 FDS groups were pooled to assess dose-dependent effects (**C**). Vaccinated animals that did not survive infection are indicated with an “x” and labelled with the animal number. *, *p* ≤ 0.05; **, *p* ≤ 0.01; ***, *p* ≤ 0.001; ****, *p* ≤ 0.0001.

Replication of rVSVΔG-MARV P3 and PHV01 was assessed by quantifying MARV GP-specific RNA in the blood of animals via RT-qPCR (Figure 3A,B). At 2 days postvaccination (DPV), the majority of vaccinated animals showed robust levels of RNA that loosely correlated with the dose level of vaccine administered. As expected, unvaccinated control animals exhibited no detectable levels of MARV GP RNA during the vaccination phase. With the exception of two guinea pigs (animals 16 and 60), the three remaining vaccinated, non-surviving guinea pigs (animals 9, 31, and 32) showed no detectable levels of MARV GP RNA postvaccination. By 27 DPV, MARV GP RNA was no longer detectable in any animal (data not shown). These data suggest that, following vaccination with rVSVΔG-MARV P3 or PHV01, most animals experience transient vaccinemia, although we did not quantify levels of infectious virus. Similar results have been observed in NHPs [12,17,24,25]. Interestingly, a few vaccinated, surviving animals had no detectable MARV GP RNA at 2 DPV (Figure 3A), suggesting that VSV vaccinemia per se may not be essential for protective immunity or that we missed the window for detecting vaccinemia by only testing a single time point. We also cannot exclude a potential role for localized VSV replication at the site of IM vaccination in eliciting a protective immune response.

To analyze the level of vaccinemia in animals immunized with the clonal PHV01 vaccines, we once again pooled the data from the PHV01 MVS and FDS medium- and high-dose groups (Figure 3C). Unsurprisingly, this analysis revealed a statistically significant difference in MARV GP RNA levels in the vaccinated versus unvaccinated animals. Moreover, it demonstrated a significant dose-dependent difference between the medium and high dose levels.

### 3.3. The Majority of Vaccinated Animals Exhibit No MARV Viremia

**Figure 4:**
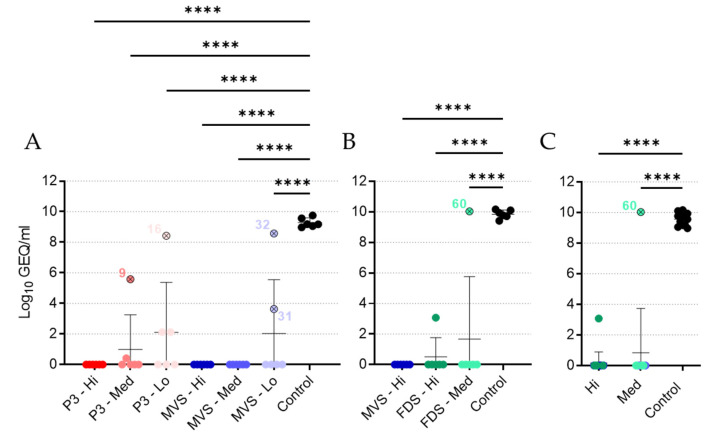
Viremia following GPA-MARV challenge. Blood samples obtained from all guinea pigs at 5 days post-infection (DPI) in Experiment #1 (**A**) or Experiment #2 (**B**) were assessed for levels of virus RNA via RT-qPCR. Data are presented as Log_10_ genome equivalents (GEQ) per milliliter, with means and standard deviations for each group depicted behind individual values for each animal. Data from the high and medium dose levels for the PHV01 MVS and PHV01 FDS groups were pooled to assess dose-dependent effects (**C**). Vaccinated animals that did not survive infection are indicated with an “x” and labelled with the animal number. Note that a technical error meant that animal 9 did not receive the full vaccine dose. All vaccinated groups showed significant differences compared to controls (****, *p* ≤ 0.0001).

In addition to the survival outcome, GPA-MARV/Ang viremia serves as an indicator of the severity of disease and, by extension, the effectiveness of the vaccines. Thus, MARV GP-specific RNA in the blood of animals was quantified via RT-qPCR. At 5 DPI, all control animals exhibited very high levels of MARV RNA in the blood, with an average of approximately 9–10 Log_10_ GEQ/mL (Figure 4A,B), indicating abundant MARV replication concomitant with severe infection. Conversely, the majority of vaccinated animals exhibited no viral RNA in the blood at 5 DPI, suggesting that vaccine-elicited immunity prevented MARV replication. Indeed, all vaccinated groups of animals showed significantly lower mean MARV RNA levels compared with the control animals. Notably, four of the five vaccinated, non-surviving guinea pigs (animals 9, 16, 32, and 60) exhibited relatively high levels of MARV RNA above ~5 Log_10_ GEQ/mL, while animal 31, which experienced a protracted disease course, had 3.6 Log_10_ GEQ/mL MARV RNA at 5 DPI. By 29 DPI, MARV RNA was undetectable in all surviving animals (data not shown), suggesting resolution or lack of MARV infection. Pooling the data from the clonal PHV01 MVS and FDS medium- and high-dose groups demonstrated a statistically significant difference in average MARV RNA levels compared to the control animals (Figure 4C). No difference was observed between the two dose levels, however, suggesting that both the medium and high dose are equally effective at preventing MARV replication.

### 3.4. Vaccinated Animals Mount a Robust Humoral Response to MARV GP

**Figure 5:**
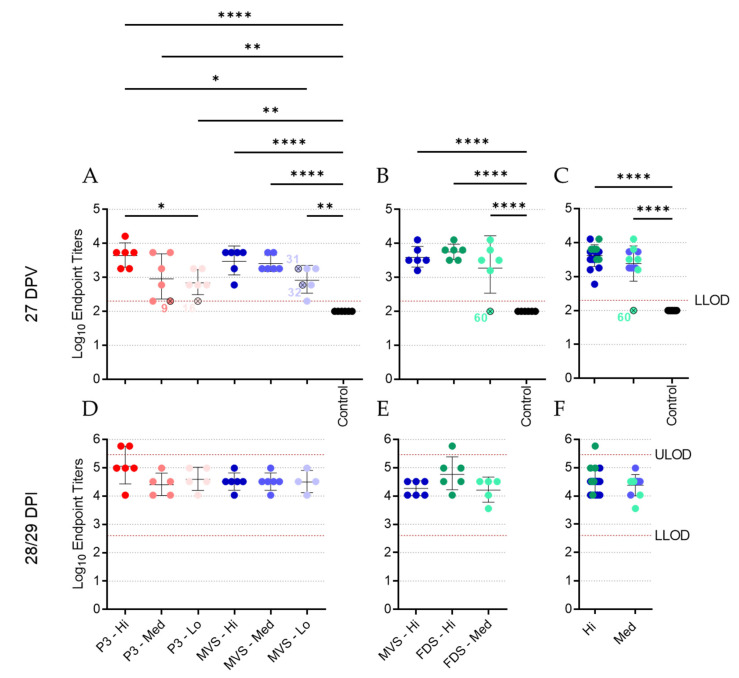
Anti-MARV GP IgG levels following vaccination and MARV challenge. Plasma samples from Experiment #1 (**A**,**D**) or serum samples from Experiment #2 (**B**,**E**) were obtained from all guinea pigs at 27 DPV (**A**,**B**) and 28 or 29 DPI (**D**,**E**) and assessed for levels of MARV GP-specific IgG via ELISA. Data are presented as endpoint titers, with geometric means and standard deviation for each group depicted behind individual values for each animal. Data from the high and medium dose levels for the PHV01 MVS and PHV01 FDS groups were pooled to assess dose-dependent effects (**C**,**F**). Vaccinated animals that did not survive infection are indicated with an “x” and labelled with the animal number. The lower limit of detection (LLOD) and upper limit of detection (ULOD) of the assays are labelled and indicated with a red dashed line. *, *p* ≤ 0.05; **, *p* ≤ 0.01; ****, *p* ≤ 0.0001.

To assess the humoral response elicited by vaccination and infection, plasma or serum samples from 27 DPV (-1 DPI) and 56 or 57 DPV (28 or 29 DPI) were evaluated by IgG ELISA (Figure 5 and Table S3). At 27 DPV, most vaccinated animals, regardless of vaccine dose level, showed high endpoint IgG titers, while all unvaccinated, control animals were IgG-negative (Figure 5A,B). Indeed, the geometric mean endpoint titers for all vaccinated groups were significantly higher than that of the unvaccinated animals. Of the vaccinated, non-surviving guinea pigs, most had low or undetectable IgG endpoint titers, with the exception of animals 31 and 32, which exhibited moderate IgG levels (Figure 5A). Pooling the data from the PHV01 MVS and FDS groups showed no significant difference in mean IgG titers between the medium and high dose levels, suggesting that both doses of vaccine elicited similarly robust immune responses (Figure 5C). Moreover, logistic regression analysis comparing IgG endpoint titers and survival for all animals immunized with PHV01 MVS or FDS revealed a 90% probability of surviving infection with an IgG endpoint titer of 1600 (~Log_10_ 3.2) (Figure S2), which was achieved in almost all vaccinated animals from the medium- and high-dose groups. At 28 or 29 DPI (56 or 57 DPV), IgG endpoint titers increased to similar levels for all surviving animals, signifying an enhancement in the immune response to MARV following infection (Figure 5D,F).

### 3.5. Vaccinated Animals Exhibit a Neutralizing Antibody Response

**Figure 6:**
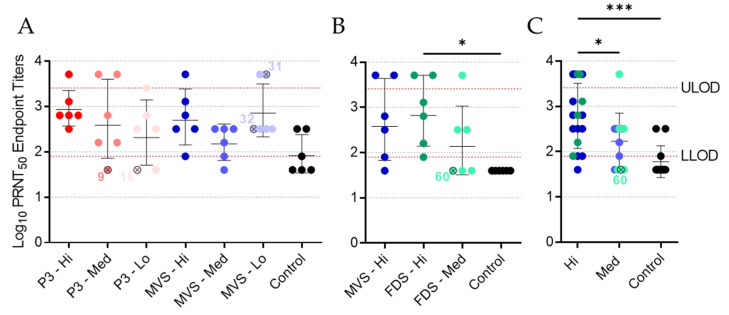
Neutralizing antibody levels following vaccination. Plasma samples from Experiment #1 (**A**) or serum samples from Experiment #2 (**B**) were obtained from all guinea pigs at 27 DPV and assessed for levels of MARV GP-specific neutralizing antibodies via PRNT_50_ assay. Data are presented as endpoint titers, with geometric means and standard deviations for each group depicted behind individual values for each animal. Data from the high and medium dose levels for the PHV01 MVS and PHV01 FDS groups were pooled to assess dose-dependent effects (**C**). Vaccinated animals that did not survive infection are indicated with an “x” and labelled with the animal number. The lower limit of detection (LLOD) and upper limit of detection (ULOD) of the assays are labelled and indicated with a red dashed line. *, *p* ≤ 0.05; ***, *p* ≤ 0.001.

Plasma or serum samples collected at 27 DPV were subjected to a PRNT assay (Figure 6 and Table S3). Animals immunized with the high dose of rVSVΔG-MARV P3 exhibited high levels of neutralizing activity, with a geometric mean PRNT_50_ endpoint titer of 905, while animals in the medium- and low-dose groups exhibited slightly lower geometric mean levels of neutralizing activity despite a much wider range of responses in individual animals (Figure 6A). Both of the non-surviving animals in the rVSVΔG-MARV P3 group (animals 9 and 16) had essentially no neutralizing response, which correlated with their overall low levels of MARV GP-specific IgG (Figure 5). Animals in the PHV01 MVS high-dose group exhibited geometric mean PRNT_50_ endpoints of 570 or 508, depending on the experiment (Figure 6A,B), with a wide range in individual neutralizing activities. While the geometric mean PRNT_50_ endpoint was lower in the PHV01 MVS medium-dose group, the neutralizing activity in the low dose group appeared quite high, with a geometric mean PRNT_50_ endpoint of 806 (Figure 6A), although this can likely be attributed to samples from two guinea pigs that exhibited very high neutralizing activity. Surprisingly, one of these guinea pigs was animal 31, which experienced a protracted disease course. Animals in the PHV01 FDS high-dose group exhibited a high level of neutralizing activity, with a geometric mean PRNT_50_ endpoint of 806, while animals in the PHV01 FDS medium-dose group mostly had lower levels, including animal 60, which lacked any apparent neutralizing activity (Figure 6B), in agreement with the absence of an IgG response (Figure 5B). Almost all of the unvaccinated control animals had no neutralizing activity, with PRNT_50_ titers falling below the lower limit of detection (Figure 6). Overall, the neutralizing activity in each of the vaccinated groups of animals appeared similar in magnitude. Pooling the data from the clonal PHV01-vaccinated animals, however, revealed that the high-dose groups had statistically significant higher geometric mean PRNT_50_ endpoint titers compared to both the control animals and the animals vaccinated with the medium dose (Figure 6C). Together, these data suggest that immunization with PHV01 elicits a potent and dose-dependent neutralizing antibody response.

Notably, three control animals did exhibit mild neutralizing activity (Figure 6A); however, we attribute these results to nonspecific inhibitory activities of the lithium–heparin that was present in the plasma samples obtained in this experiment. In the second experiment (Figure 6B), we obtained serum samples devoid of lithium–heparin, which did not possess the same nonspecific inhibitory activity (cf. Figure 6A,B). Accordingly, the endpoint titers presented in Figure 6A may be artificially elevated, particularly at the lower dilutions.

## 4. Discussion

A safe and effective vaccine capable of preventing MVD is urgently needed. Although MARV has been responsible for just over a dozen outbreaks over the past five decades, it remains a potentially serious threat to global public health, capable of causing severe disease and unpredictable epidemics. The recent report of MVD in Guinea exemplifies the ongoing threat that this virus poses to Africa, and circulation of the virus in bats throughout the continent [26,27,28,29,30,31], including in West Africa [32], further underscores the risk to public health. Moreover, past instances of travelers returning home with MVD demonstrate the possibility of international spread [33,34]. While many experimental MARV vaccines have been developed over the last several years, only a few candidates have been evaluated in small Phase I clinical trials and none have been approved for use in humans [8,35].

In this study, we evaluated the protective efficacy of the live-attenuated PHV01 candidate vaccine, which expresses the MARV/Ang GP in a recombinant VSV backbone in place of the VSV G gene. Importantly, we evaluated both uncloned (rVSVΔG-MARV P3) and biologically cloned (PHV01 MVS and PHV01 FDS) viruses at different passage levels in an effort to not only confirm efficacy against GPA-MARV/Ang in the guinea pig model of infection, but also to confirm the reproducible efficacy of a clonally derived vaccine candidate, PHV01 FDS, produced via a manufacturing process representative of the GMP process that will be used to produce clinical material. Our results demonstrate that both PHV01 FDS and PHV01 MVS are highly effective, offering 100% protection at the highest dose levels tested and ~97% protection over the medium and high dose levels combined. Together, PHV01 MVS and FDS elicited significant IgG and neutralizing antibody responses that reduced or eliminated MARV replication in most vaccinated animals. These conclusions were not unexpected given previous studies performed with rVSVΔG-MARV/Ang and rVSVΔG-MARV/Mus in NHPs [12,13,17,18,19,24,25]. Nevertheless, this study represents the first published evaluation of an rVSV-based MARV vaccine in a rodent, and it attests to the predictive value of the MARV guinea pig model in countermeasure evaluation.

The humoral immune response is thought to be essential to the immune protection conferred against EBOV by the rVSVΔG-EBOV vaccine [36], and the same appears true for PHV01. All prior evaluations of rVSVΔG-MARV in NHPs demonstrated moderate to high levels of MARV GP-specific IgG response following vaccination [12,13,17,18,24,25], and the present study recapitulates these results in the guinea pig model. Almost all the vaccinated guinea pigs exhibited high levels of IgG at 27 DPV, and this was particularly evident in the animals that received the high and medium doses. Neutralizing activity was also detected in plasma/serum samples from most vaccinated animals, although the individual titers varied widely within each group and some surviving animals had no detectable levels of neutralizing activity. Interestingly, high levels of neutralizing antibodies have not been consistently identified in NHPs vaccinated with rVSVΔG-MARV [12,13,17], suggesting that non-neutralizing antibodies may contribute to immunity through various other effector functions [37]. Work with EBOV GP-based vaccines using the human parainfluenza virus or adenovirus/MVA platforms has demonstrated that Fc-mediated antibody effector functions contribute important immune protection in NHPs and humans, respectively [38,39]. Likewise, survivors of Ebola virus disease have been shown to develop polyfunctional antibodies capable of mediating a variety of Fc-mediated effector functions [40]. Whether similar immune responses are elicited by PHV01 is currently unclear, but a recent study evaluating a different rVSVΔG-MARV/Ang vaccine in NHPs corroborated the induction of strong humoral immunity and described extensive transcriptional changes associated with innate immune activation and B- and T-cell proliferation, suggesting potential roles for other immune components [18].

It is difficult to conclude whether the immunity elicited by PHV01 in this study was sterilizing, since we only quantified MARV infection via RT-qPCR at a single time point (i.e., 5 DPI) during acute disease. Nevertheless, no MARV RNA was detected in the vast majority of animals that received the high and medium doses of vaccine. Within these groups, virus RNA was undetectable in all but three animals—two of which ended up succumbing to disease. One of the non-survivors (animal 9; rVSV-MARV P3 medium-dose group) did not receive the full dose of vaccine, while the other non-survivor (animal 60; PHV01 FDS medium-dose group) may not have responded to vaccination at all, since no IgG or neutralizing antibody response was detected and only very low levels of vaccinemia were detected after immunization. The third animal—a survivor from the PHV01 FDS high-dose group—exhibited low levels of MARV RNA, although it is unclear how this relatively low RNA level correlates with infectious virus load. In any case, these results are in line with what has been observed in NHPs, where infectious MARV has never been reported in vaccinated animals [12,13,17,18,24,25]. At the low vaccine doses, we did detect high levels of MARV RNA in a few guinea pigs, all of which eventually succumbed to disease. Animals 16 and 32 exhibited low levels of MARV GP-specific IgG at 27 DPV, and either no or low levels of neutralizing antibodies, respectively, suggesting that the immune response was not sufficient to prevent disease. Conversely, animal 31 exhibited moderate levels of IgG and high levels of neutralizing activity despite eventually meeting the humane endpoint at 14 DPI, a week after all other non-survivors. It is not clear what caused the protracted disease in this animal, but we suspect that the outbred nature of these guinea pigs may introduce host-specific factors capable of influencing the animal’s response to vaccination or MARV infection. Alternatively, the virus may have acquired mutations that permitted it to escape the immune response elicited by vaccination.

In summary, our study demonstrated for the first time the protective efficacy of a VSV-based MARV GP vaccine in guinea pigs through a single-dose immunization. rVSVΔG-MARV P3 and PHV01 provided high survival rates against homologous MARV/Ang challenge, protecting 100% of the animals at the highest dose level tested and offering significant protection even at very low dose levels. In addition, our findings in guinea pigs aligned closely with the findings from NHP studies, including a recent study demonstrating PHV01 confers 100% protection against death, clinical signs of disease, and characteristic changes in clinical chemistry parameters in cynomolgus macaques challenged with a uniformly lethal dose of MARV/Ang (Shannan Rossi, personal communications). In light of the results presented here, this NHP study further highlights the utility of the guinea pig model of MARV infection as a bridge to NHPs.

Importantly, this study also specifically confirms the efficacy of the selected PHV01 FDS clonal vaccine candidate, with no significant difference observed in the degree of protection afforded by the uncloned and cloned materials. Furthermore, these findings validate the fidelity of the production process. PHV01 FDS was produced via a manufacturing process representative of the GMP process being used to produce vaccine material for clinical trials and regulated nonclinical testing. As demonstrated here, this process preserved MARV/Ang GP and its critical functional attributes through multiple passage levels. Notably, although PHV01 FDS, itself, was not sequence-confirmed, viruses from multiple passage levels preceding PHV01 FDS (including PHV01 MVS) displayed remarkable sequence fidelity, as did virus that was passaged three times beyond PHV01 MVS (Figure S1), highlighting the stability of the rVSV genome. Moreover, the immune responses to all vaccine materials were similar, suggesting no impactful divergence among the three viruses. Even though the present study was not conducted under GLP conditions, the results bridge the efficacy observed in previous NHP studies using research-grade vaccine candidates with efficacy observed in a well-characterized guinea pig model using a vaccine candidate ready for clinical production. Together, these results provide additional evidence to advance preclinical and clinical development of this important human vaccine candidate.

## Data Availability

Data are presented in the manuscript. Raw data are available from the corresponding author upon reasonable request.

## Supplementary Materials

The following supporting information can be downloaded at: https://www.mdpi.com/article/10.3390/vaccines10071004/s1, Figure S1: Passage history of rVSVΔG-MARV; Figure S2: Simple Logistic Regression Analysis Comparing IgG Endpoint Titers with Survival; Table S1: Vaccine Efficacy; Table S2: PHV01 Vaccine Efficacy; Table S3: Antibody Responses at 27 DPV.
