# Supplementary material for: A Cloned Recombinant Vesicular Stomatitis Virus-Vectored Marburg Vaccine, PHV01, Protects Guinea Pigs from Lethal Marburg Virus Disease
Source: Vaccines (Basel). 2022 Jun 23;10(7):1004. doi: 10.3390/vaccines10071004 (PMC9324024; doi:10.3390/vaccines10071004)
Supplement: Supplementary file 1 [file vaccines-10-01004-s001.zip › vaccines-1756800-supplementary.pdf]

vaccines

MDPI

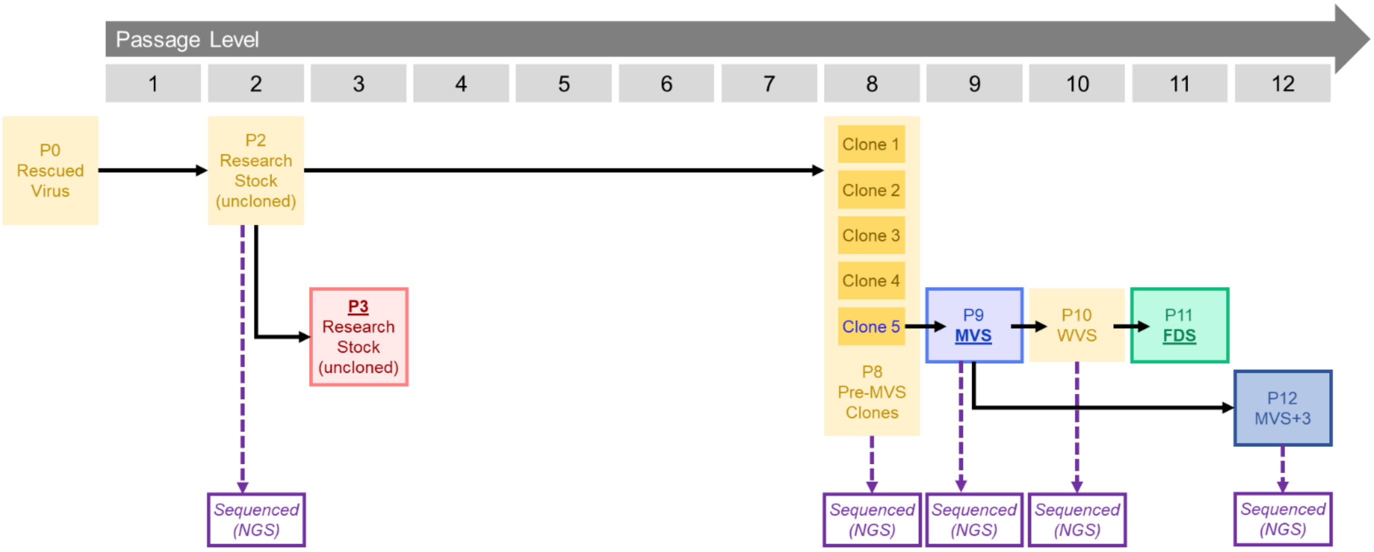

Figure S1. Passage history of rVSVΔG-MARV. rVSVΔG-MARV was initially generated by reverse genetics "rescue" from cells transfected with plasmids encoding the VSV polymerase complex, as well as a recombinant VSV genome expressing MARV/Ang GP in place of VSV G. The rescued virus is designated passage level 0 (P0). An uncloned research stock of the virus was amplified to P2. The P2 stock was subsequently amplified and plaque purified to generate premaster viral seeds at P8. A single P8 clone was chosen and grown under GMP conditions to P9 to generate PHV01 Master Viral Seed (PHV01 MVS). PHV01 MVS was grown under GMP conditions to P10 to generate a PHV01 Working Viral Seed (PHV01 WVS), which was then passaged to P11 to generate PHV01 Formulated Drug Substance (PHV01 FDS). rVSVΔG-MARV P3 was generated from the P2 stock through an independent passage. PHV01 MVS was passaged three additional times (MVS +3) to P12 and sequenced to confirm the stability of the virus genome. Indeed, using the sequence of the full-length genome plasmid as a reference, next-generation sequencing (NGS) confirmed the sequence identity of the indicated viruses at multiple different passage levels (i.e., P2, P8, P9, P10, and P12), demonstrating the stability of the rVSV genome. The present study evaluated the protective efficacy of P3 (red box), PHV01 MVS (light blue box), and PHV01 FDS (green box).

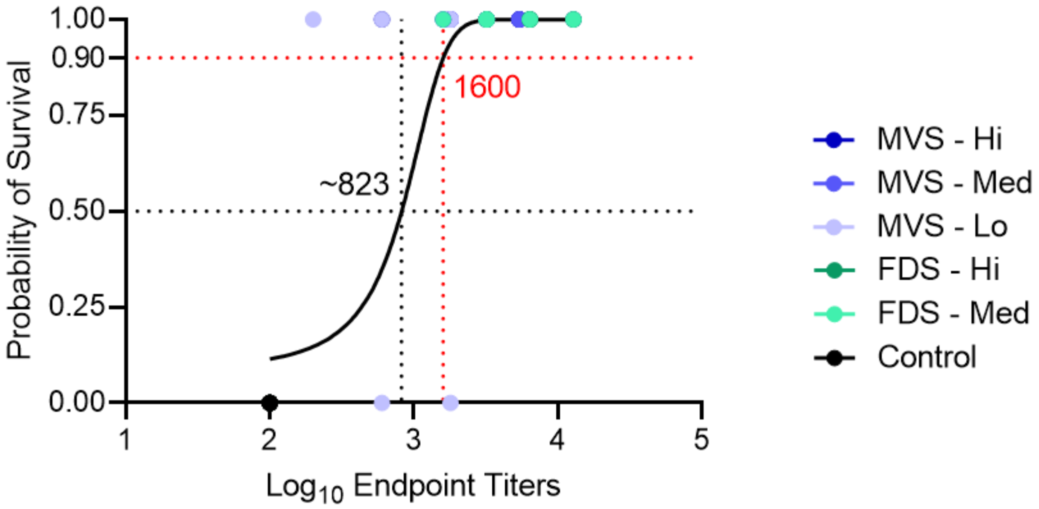

Figure S2. Simple logistic regression analysis comparing IgG endpoint titers with survival. IgG endpoint titers from animals vaccinated with PHV01 MVS or FDS were plotted against survival outcome following GPA-MARV challenge and analyzed by logistic regression. Results revealed a 50% probability of surviving with an IgG endpoint titer of ~823 (~2.9 Log_{10}) and a 90% probability of surviving with an IgG endpoint titer of 1600 (~3.2 Log_{10}). Tjur's R-squared, 0.7301; Cox-Snell's R-squared, 0.5707.

Table S1. Vaccine efficacy.

**Exp.**	Vaccine	Vaccine Dose		Survival	
		Category	PFU	Number[1]	%
**#1**	P3	Hi	2E6	6 / 6	100
		Med	2E4	5 / 6	83
		Lo	2E2	5 / 6	83
	MVS	Hi	2E6	6 / 6	100
		Med	2E4	6 / 6	100
		Lo	2E2	4 / 6	67
	Control	-	-	0 / 6	0
#2	MVS	Hi	2E6	6 / 6	100
	FDS	Hi	2E6	6 / 6	100
		Med	2E4	5 / 6	83
	Control	-	-	0 / 6	0
		**Total P3 + MVS + FDS**		49 / 54	91

[1]Number of survivors divided by total number.

Table S2. PHV01 vaccine efficacy.

N	PHV01 MVS + FDS		Survival	
	Dose	PFU	Number[1]	%
18	Hi	2E6	18 / 18	100
12	Med	2E4	11 / 12	92
		**Total**	**29 / 30**	**97**

[1]Number of survivors divided by total number.

Table S3. Antibody responses at 27 DPV.

Sample Type	Vaccine	Vaccine Dose		IgG		$PRNT_{50}$	
		Category	PFU	GMT[1]	Response[2]	GMT	Response
Plasma (Exp. #1)	P3	Hi	2E6	4496	6 / 6 (100%)	905	6 / 6 (100%)
		Med	2E4	1039	6 / 6 (100%)	508	5 / 6 (83%)
		Lo	2E2	721	6 / 6 (100%)	254	4 / 6 (67%)
	MVS	Hi	2E6	3118	6 / 6 (100%)	570	6 / 6 (100%)
		Med	2E4	2596	6 / 6 (100%)	160	5 / 6 (83%)
		Lo	2E2	865	6 / 6 (100%)	806	6 / 6 (100%)
	Control	-	-	<LLOD[3]	0 / 6 (0%)	90	3 / 6 (50%)
Serum (Exp. #2)	MVS	Hi	2E6	4032	6 / 6 (100%)	508	5 / 6 (83%)
	FDS	Hi	2E6	5702	6 / 6 (100%)	806	6 / 6 (100%)
		Med	2E4	2263	5 / 6 (100%)	180	3 / 6 (50%)
	Control	-	-	<LLOD	0 / 6 (0%)	<LLOD	0 / 6 (0%)

[1]GMT, geometric mean titre; [2]response, number of animals with IgG or $PRNT_{50}$ endpoint titers at or above LLOD divided by total number of animals; [3]LLOD, lower limit of detection.
